# Large-science facilities must continue to add value to the scientific community

**DOI:** 10.1107/S2052252519011709

**Published:** 2019-08-29

**Authors:** D. N. Argyriou

**Affiliations:** a European Spallation Source ERIC, PO Box 176, SE-221 00 Lund, Sweden

**Keywords:** large-science facilities, editorial

## Abstract

With the technological development of affordable and impactful laboratory-based tools and their ever-growing scientific scope, large-science facilities need to review their strategies in order to continue to add value for their scientific communities.

The main arguments that drive investment in large-science facilities are the new scientific insights that will be gained by significantly enhanced technical performance. The case for free electron lasers (FELs), for example, is made on unprecedented generation of photons with a unique time structure, which allow researchers to see inside single molecules. Similarly, snappy ‘*science cases*’ are made for both the Large Hadron Collider at CERN and the European Spallation Source in Lund, Sweden. However, with highly visible costs to run large-science facilities, university researchers, and rightly so, do challenge these commitments, with the often-heard argument of ‘*give me the money instead and I will spend it on real science!*’. While university funding should be a priority, the real question being asked is ‘*Do large-science facilities provide value for making progress in science?’.* This question is now becoming even more pressing as new advances provide highly impactful tools for scientific discovery, at a price that most well-funded universities can afford.

Advanced technical performance provided by large-science facilities alone is not sufficient to either justify the investment or sustain substantial long-term operational costs. The true value of these facilities is harnessed over many decades by an engaged scientific community, especially when the facility becomes an integral component of that community. That impact is clear when viewing the stream of key scientific results they have provided over the decades and the support they have contributed for breakthrough Nobel-Prize-winning work (such as work on soft-mode transitions, spin excitation in superconductors, glass transitions, magnetic monopoles, protein structures and fast dynamics). Their impact also goes beyond the scientific results alone by often developing technologies that have wide societal impact (html, capacitive displays, modern cryostats and many others).

Value is an abstract concept and hard to generalize as it varies between researchers and scientific communities. For some, it may be as simple as the opportunity for a nearby ski-trip after attending an experiment at a facility. For most, there are the added benefits of unique measurements that are not charged to your research grant. A user of a large-science facility gets a lot of added value in terms of free support; this can include software and experimental support, consumables such as liquid He, sometimes paid travel, opportunities to network and attend important seminars, cost sharing on students *etc*. Indeed, some universities acknowledge this added value by treating accepted beamtime proposals as grants into their departments. And, yes, these hard to come by measurements can be a career launch pad for many young scientists.

While this large-science business model of providing unique scientific capability combined with high-quality scientific and technical support has worked for many decades, it is now challenged on two fronts. The first front comes from continual technical innovation and development, which can deliver high-profile impactful science at an affordable price to most well-funded universities. Cryo-electron microscopy is one example, but others exist such as laser-ARPES (a technique usually performed at synchrotrons), magnetic imaging using Lorentz lenses in electron microscopes (a technique that can compete with experiments performed at neutron sources), quantum sensing and computing. New laboratory X-ray equipment can now routinely provide third-generation synchrotron performance. Investments in these new technologies runs at a broad but affordable price scale of 500k to 15M USD. The advantages are clear, potentially highly impactful scientific results, easy access for local researchers, unique capabilities and ability to control future scientific and technical directions. The argument that this competitive advantage is lost if everyone has access to such capabilities misses the point that the advantage often lies in timing of research, the sample examined and the skill of the researchers. The actual case to justify these investments by universities is that, if they do not invest in these powerful and affordable tools, they will not be able to attract top talent or remain scientifically competitive.

The second challenge to the facility business models comes from their own success, in the form of mission or scope creep. At facility reviews, the most hotly discussed areas after the number of beam days provided to users and the number of high-impact publications, are all value-added science support items such as data analysis, support for sample environment *etc*. It is natural, of course, given the position of many large-scale facilities at the nexus of scientific and technological advances, to try to drive innovation in many key areas that enable new science. Facilities also benefit from resources, engineering expertise and project management skills to drive that technological innovation. However, in order to remain competitive and add value, the potential for upward scope creep is clearly evident. This can include sample preparation and characterization laboratories, deuteration facilities, high-pressure facilities, high-performance computing infra-structure – the list grows continually. The diversity and size of scope that adds value, and is demanded by the community, is significant and facilities are stretched to their very limits to satisfy these demands.

With the technological development of affordable and impactful laboratory-based tools and their ever-growing scientific scope, large-science facilities need to review their strategies in order to continue to add value for their scientific communities. To ensure success, facilities will need to be fully cognizant of the scientific and technological developments that are taking place outside their own domain. Competitive analysis with techniques available at an affordable price to universities will be needed in order to drive priorities and new investments. New strategies to remain competitive will likely challenge some established fields that have had a long-term presence in large-scale facilities, but perhaps are best served now by other means. Creating that headroom to allow for new innovations and investment for these new strategies, is essential in order to develop new opportunities that will continue to add value to the scientific community for the next decades. What is also essential is enhancing and possibly rethinking engagement with scientific communities, perhaps even giving these communities a greater role in the value-chain of large-science facilities.

